# Genetic Variants of the α-Synuclein Gene *SNCA* Are Associated with Multiple System Atrophy

**DOI:** 10.1371/journal.pone.0007114

**Published:** 2009-09-22

**Authors:** Ammar Al-Chalabi, Alexandra Dürr, Nicholas W. Wood, Michael H. Parkinson, Agnes Camuzat, Jean-Sébastien Hulot, Karen E. Morrison, Alan Renton, Sigurd D. Sussmuth, Bernhard G. Landwehrmeyer, Albert Ludolph, Yves Agid, Alexis Brice, P. Nigel Leigh, Gilbert Bensimon, for the NNIPPS Genetic Study Group

**Affiliations:** 1 MRC Centre for Neurodegeneration Research, King's College London, Department of Clinical Neuroscience, Institute of Psychiatry, and NIHR Biomedical Research Centre, London, United Kingdom; 2 INSERM, UMRS975 (formerly UMR_S679), Paris, France; 3 AP-HP, Hôpital Pitié-Salpêtrière, Department of Genetics and Cytogenetics, Paris, France; 4 Department of Molecular Neuroscience, Institute of Neurology, University College London, London, United Kingdom; 5 Département Pharmacologie Clinique, Centre Hospitalo-Universitaire de la Pitié-Salpétrière, Assistance Publique Hôpitaux de Paris & UPMC Univ. Paris 06, UMR 7087, Paris, France; 6 Department of Clinical Neurosciences, School of Clinical and Experimental Medicine, University of Birmingham, Birmingham, United Kingdom; 7 Abteilung Neurologie, Universität Ulm, Ulm, Germany; 8 Centre d'Investigation Clinique, Hôpital de la Pitié-Salpêtrière, Paris, France; VU University Medical Center and Center for Neurogenomics and Cognitive Research; VU University Medical Center and VU University, Amsterdam, the Netherlands, Netherlands

## Abstract

**Background:**

Multiple system atrophy (MSA) is a progressive neurodegenerative disorder characterized by parkinsonism, cerebellar ataxia and autonomic dysfunction. Pathogenic mechanisms remain obscure but the neuropathological hallmark is the presence of α-synuclein-immunoreactive glial cytoplasmic inclusions. Genetic variants of the α-synuclein gene, *SNCA*, are thus strong candidates for genetic association with MSA. One follow-up to a genome-wide association of Parkinson's disease has identified association of a SNP in *SNCA* with MSA.

**Methodology/Findings:**

We evaluated 32 SNPs in the *SNCA* gene in a European population of 239 cases and 617 controls recruited as part of the Neuroprotection and Natural History in Parkinson Plus Syndromes (NNIPPS) study. We used 161 independently collected samples for replication. Two *SNCA* SNPs showed association with MSA: rs3822086 (*P* = 0.0044), and rs3775444 (*P* = 0.012), although only the first survived correction for multiple testing. In the MSA-C subgroup the association strengthened despite more than halving the number of cases: rs3822086 *P* = 0.0024, OR 2.153, (95% CI 1.3–3.6); rs3775444 *P* = 0.0017, OR 4.386 (95% CI 1.6–11.7). A 7-SNP haplotype incorporating three SNPs either side of rs3822086 strengthened the association with MSA-C further (best haplotype, *P* = 8.7×10^−4^). The association with rs3822086 was replicated in the independent samples (*P* = 0.035).

**Conclusions/Significance:**

We report a genetic association between MSA and α-synuclein which has replicated in independent samples. The strongest association is with the cerebellar subtype of MSA.

**Trial Registration:**

ClinicalTrials.gov NCT00211224. [NCT00211224]

## Introduction

Multiple system atrophy (MSA) is a rare progressive neurodegenerative disease characterized by parkinsonism, cerebellar dysfunction and dysautonomia corresponding to previous diagnostic appellations of striatonigral degeneration, olivopontocerebellar atrophy and Shy-Drager syndrome. Estimates of age-adjusted prevalence vary between 1.9 and 4.4 per 100,000 persons although this may represent an underestimate as post-mortem studies have suggested that as many as 5% of cases of clinically diagnosed Parkinson's disease may in fact have MSA. The converse is also true however, and in those recruited as part of the Neuroprotection and Natural History in Parkinson's Plus Syndromes (NNIPPS) study, about 7% of patients diagnosed with MSA at the start had a different diagnosis by the end [1].

Pathologically, the hallmark of MSA is α-synuclein-immunoreactive glial cytoplasmic inclusions in oligodendrocytes throughout the brain, associated with neuronal loss, loss of myelin, astrocytosis and a marked microglial reaction most prominent in brain regions involved in motor and supraspinal autonomic control. The presence of α-synuclein-immunoreactive pathology, which places MSA amongst the broad category of synucleinopathies including Parkinson's disease and dementia with Lewy bodies, suggests that an abnormality of α-synuclein may play a role in the mechanisms of cell death in MSA.

MSA is generally considered a sporadic condition. Indeed, the diagnostic consensus criteria for MSA specifically mention a family history of parkinsonism or ataxia as a feature not supportive of the diagnosis [2]. Nonetheless, a few apparently familial cases have been described [3]–[8]. There have been few studies on genetic sequence variants in sporadic MSA [9]–[20] and no environmental risk factors have been consistently identified in large samples. A genetic association has now been identified between a gene variant in the α-synuclein gene (*SNCA*, OMIM accession *163890; Ensembl ENSG00000145335) and MSA [21].

We examined the association between *SNCA* variants and MSA using the DNA Bank of the NNIPPS cohort [1]. This cohort has a nearly 50% autopsy rate for MSA so that the criteria for diagnosis can be validated pathologically, and in individual cases we can be certain of the diagnosis. The NNIPPS diagnostic criteria for MSA (Table 1) show excellent convergent validity with the investigators' assessment of diagnostic probability (point-biserial correlation: MSA rpb = 0.93, P<0.0001), and excellent predictive validity against histopathology [sensitivity and specificity (95% CI); 0.96 (0.88–0.99) and 0.91 (0.86–0.93)] [1], so the sample is likely to represent a more highly homogeneous diagnostic group than is usually possible. For replication of results, we analyzed a population of 78 pathologically verified cases from the Institute of Neurology Brain Bank and 83 clinically defined samples from a Parisian study (Table 2). We found that genetic variants of *SNCA* were associated with MSA and more specifically, with the subset of people who had cerebellar signs.

**Table 1:** Summary of diagnostic criteria for MSA used in the NNIPPS study.

Inclusion criteria		Exclusion criteria
All of:	And at least one of:	Any of:
Akinetic-rigid syndrome	Symptomatic autonomic dysfunction	Evidence of another neurological diagnosis
Age at onset at least 30 years old	Cerebellar ataxia	Evidence of a different cause for an akinetic-rigid syndrome (eg drug side effect)
Disease duration 12 months to 8 years	Postural instability or falls within 3 years of onset	Dementia
	Pyramidal signs	

**Table 2:** Demographic features of the samples used.

Sample type	*N*	Age at onset (SD)	Age at sampling (SD)	M:F ratio	Proportion MSA-C
**NNIPPS samples**					
MSA all	239	58.0 (8.43)	62.8 (8.25)	0.56	0.44
Pathological diagnosis	44	57.0 (8.41)	61.9 (7.94)		
Clinical diagnosis	195	58.2 (8.44)	63.1 (8.32)		
Controls	617	N/A	61.0 (12.9)	0.47	N/A
**Replication samples**					
Paris Sample Bank	83	54.7 (8.67)		0.65	
IoN Brain Bank	78	56.5 (10.0)		0.47	

## Methods

The protocol for this trial and supporting CONSORT checklist are published elsewhere [1].

### Ethics Statement

Patients gave informed written consent prior to inclusion in the trial. Additional consent was obtained both for DNA sampling and post-mortem brain tissue donation. The protocol was approved by the Ethics Committees/Institutional Review Boards of each coordinating centre in the three participating countries. The trial was conducted according to International standards of Good Clinical Practice-(ICH guidelines and the Helsinki Declaration).

### Study Subjects

Subjects were recruited as part of the Neuroprotection and Natural History in Parkinson's Plus Syndromes (NNIPPS) study using the NNIPPS diagnostic criteria which have been described elsewhere together with the trial design, population description and treatment results [1] (Table 1). The 239 cases and 617 controls in this study were of European ancestry and matched for age (MSA mean age at sampling 62.84, control mean age 61.03) (Table 2). The clinical diagnosis at the study end was used to define MSA unless a pathological diagnosis was available. For exploratory sub-group analysis, patients were stratified as MSA-P or MSA-C based on the absence or presence of cerebellar signs at entry. 11 cases and 14 controls had a low genotyping rate (<90%) and were excluded from further analysis. 101 of the remaining cases were in the MSA-C subgroup. 251 patients with progressive supranuclear palsy (PSP) recruited within the same study were also tested for MSA-associated SNPs to exclude a recruitment bias as the explanation for any findings, as PSP is not a synucleinopathy and we would not therefore expect an MSA-associated *SNCA* SNP to be associated with PSP.

The NNIPPS study was an academic-led, multi-centre (UK, France, Germany), phase III trial of riluzole in MSA and progressive supranuclear palsy. Controls were recruited in the UK from the neurologically normal spouses of participating patients. In France and Germany, the controls were taken from existing DNA banks of neurologically normal controls.

### Replication study samples

The 78 MSA samples from the Institute of Neurology Brain Bank (Table 2) fulfilled the consensus criteria for pathologically proven MSA [2]. All were of UK ancestry. Of these, 68% had cerebellar signs. Samples were derived from blood.

The MSA samples from Paris (Table 2) were collected consecutively as part of a study on atypical Parkinsonism and cerebellar ataxia. There were 44 cases with Probable MSA and 44 with Possible MSA as defined by the consensus criteria [2]. Of 88 samples, five were not of European ancestry and were therefore excluded from further analysis. Of these, 85% had cerebellar signs as the clinic specializes in cerebellar disease. Samples were derived from blood.

### Genotyping

**Figure 1:**
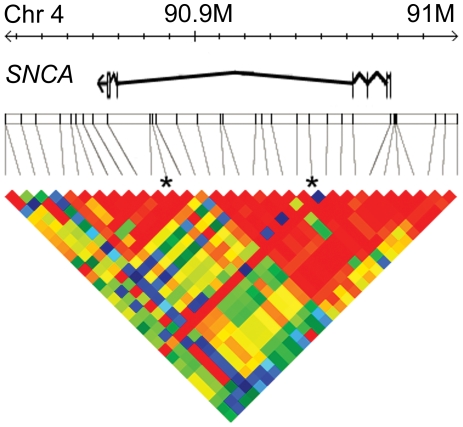
The genetic architecture of the *SNCA* gene and the markers genotyped. The base pair position along chromosome 4 is shown by the top ruler. The position of the α-synuclein gene is shown in the next row down, with exons represented by vertical lines and introns by lines connecting them. The relative positions of the genotyped markers are shown below, with vertical lines connecting position to the linkage disequilibrium map. Asterisks show the two markers demonstrating association in MSA-C. Only the left-most marker showed association with MSA as a whole. (For a list of markers genotyped and *P*-values, see Table 3). The coloured triangle is the linkage disequilibrium heat map showing the strength of association between pairs of markers as measured by D′. Red is high and blue low with the other colours intermediate. The two associated SNPs are in different linkage disequilibrium blocks and are not in linkage disequilibrium with each other.

32 SNPs in and around *SNCA* were selected for association testing with MSA (Figure 1, Table 3). In addition, we genotyped the multi-allelic microsatellite repeat known as NACP-Rep1 which is situated within the promoter region ∼10 kbp upstream of the translational start point of *SNCA* at chromosome 4q21, using an ABI3130XL Genotyper (Applied Biosystems) and Genotyper v4.0 software. This complex microsatellite with sequence (TC)_10–11_TT(TC)_8–11_(TA)_7–9_(CA)_10–11_ has been variably associated with PD [22], [23] but not MSA [12], although the numbers studied were small. There are five common alleles, each differing in size by two nucleotides, the greatest variability being in the CA portion with subsidiary variability in the (TC)_8–11_(TA)_7–9_ portion. However, *ex vivo* functional analysis suggests that the overall length of the microsatellite rather than its sequence, determines transcriptional regulation and hence *SNCA* gene expression [24]. We numbered the alleles from 1 to 5 in order of increasing size.

**Table 3:** Results for *SNCA* SNPs.

SNP	Counts Controls	Counts MSA	HWE *P*-value (controls)	Missingness *P*-value	Association *P*-value MSA	Permutation *P*-value MSA	Replication study counts	Replication *P*-value MSA	Association *P*-value MSA-C	7-SNP haplotype *P*-value with MSA-C
rs3857047	12/152/437	3/62/162	0.8712	1	0.8184	1				
rs356229	89/260/253	28/102/98	0.1127	1	0.539	1				
rs3857049	0/17/586	0/9/218	1	0.2744	0.33	0.9963				
rs3906628	4/119/480	2/51/175	0.3833	1	0.305	0.9888				
rs356180	57/253/293	18/95/114	0.8475	0.2744	0.5286	1				
rs356169	66/258/273	24/92/109	0.7097	0.7117	0.5669	1				
rs2572323	53/249/295	18/95/112	1	0.7117	0.78	1				
*rs356219	75/268/257	28/111/89	0.7191	0.5658	0.5625	0.9783				
rs356220	64/221/212	28/110/89	0.6225	4.73×10^−15^	Excluded	Excluded				
*rs356165	76/270/253	28/110/88	0.7883	0.6683	0.6281	0.9992				
*rs356204	112/293/197	44/115/69	0.8681	1	0.619	0.9998				
rs3822086	3/60/540	0/43/185	0.4124	1	0.004448	0.0479	2/24/135	0.0347	0.002403	Best: 8.70×10^−4^, omnibus 0.029
*rs2356203	76/275/251	28/111/89	1	1	0.7206	0.9998				
*rs356168	113/288/197	44/114/67	0.6769	0.6909	0.5523	0.9849				
*rs356199	51/232/317	17/86/125	0.3609	0.5658	0.5014	1				
rs356188	23/194/383	6/82/139	0.8986	1	0.6171	1				
rs356187	53/230/319	19/83/125	0.2242	0.4737	0.5715	1				
rs356186	22/181/392	4/78/143	0.8935	1	0.8487	1				
rs2737033	53/231/317	20/83/125	0.2657	1	0.6098	1				
rs2737029	85/273/244	36/111/81	0.5402	1	0.2387	0.9369				
rs2583959	44/231/316	16/87/124	0.835	0.1282	0.6785	1				
rs3775444	0/12/587	0/11/217	1	0.5797	0.01191	0.1807			0.001682	0.0017
rs2619369	0/31/572	0/17/211	1	1	0.2799	0.9908				
rs1812923	137/295/170	44/126/57	0.683	0.4737	0.8877	1				
intron4_a66g	145/279/176	45/122/60	0.1017	1	0.8587	1				
rs2301135	149/278/173	49/117/56	0.08575	0.01562	0.8361	1				
rs2619364	46/229/327	16/84/128	0.5315	1	0.6061	1				
rs2583987	46/229/325	15/84/128	0.5321	1	0.4829	0.9999				
rs2583988	46/224/327	16/84/128	0.4	0.1963	0.6633	1				
REP-1**	310/838/58/0	135/306/34/1	0.9219	N/A	0.042	0.094				
rs1372525	151/284/168	52/116/56	0.1659	0.005559	0.7551	1				
rs2737026	28/212/360	9/90/129	0.7245	0.5658	0.4652	1				
rs2736994	26/195/380	6/83/139	0.9005	1	0.8326	1				

* Markers included in the 6-SNP tagging haplotype correlating with the genotype at rs3822086, and which were added to rs3822086 to form the 7-SNP haplotype for association.

** Results for the microsatellite marker Rep-1 are given as allele counts rather than genotypes, and the permutation *P*-value is for permutation within allelic groupings only.

For the NNIPPS samples, DNA was extracted from venous blood samples collected into Li-heparin tubes by standard methods. SNPs were genotyped independently of the study group and blinded to case-control status by KBiosciences (Hoddesdon Herts, EN11 0EX, UK; www.kbioscience.co.uk) using a patent-protected competitive allele specific PCR system (KASPar) and Taqman™.

The Institute of Neurology pathological samples were genotyped for rs3822086 using Taqman™ assay (Applied Biosystems, Forster City, CA, USA).

The 83 Paris samples were genotyped for the single nucleotide polymorphism rs3822086 by sequencing in a single direction using the BigDye Terminator Cycle Sequencing kit v3.1 (Applied Biosystems, Forster City, CA, USA). Sequences were analysed using Seqscape v2.5 software.

### Statistical Analysis

#### Quality control

Tests for Hardy-Weinberg equilibrium were performed for each SNP in controls using Fisher's exact test. A *P*-value<0.05 led to exclusion from further analysis. Tests for systematically missing genotypes in either cases or controls were performed to identify sample selection or genotyping problems preferentially affecting either group. Tests for missingness by genotypic status were also performed to identify errors relating to difficulty calling certain genotypes. Associated SNPs were further tested by looking for association in a tagging haplotype that excluded the associated SNP to reduce the likelihood of an artefactual association resulting from genotyping.

#### Power

Assuming a lifetime MSA risk of 1 in 1000, and for a *P*-value close to the permutation corrected threshold of 0.05 (alpha = 0.005), there is 80% power to detect an odds ratio of 1.8 for a minor allele frequency (MAF) of 0.1, 1.6 for MAF 0.2 and 1.5 for MAF>0.2.

#### Imputation

Genotypes for SNPs genotyped in the CEU HapMap sample but not in this study were imputed using IMPUTE [25] with a threshold of 0.9 and default values for other parameters. Subsequent analysis was performed in PLINK.

#### Association testing

SNP analyses were performed in PLINK (http://pngu.mgh.harvard.edu/purcell/plink/) [26]. Samples were tested for allelic association using the Mantel-Haenszel chi-squared test, stratifying by country of origin. Odds ratios were tested for homogeneity between countries using the Breslow-Day test. Association testing by Pearson chi-squared test was performed for the replication study to maximise numbers and therefore statistical power. Controls were those used in the primary analysis.

Multiple testing correction was performed using the Max(T) permutation procedure implemented in PLINK permuted within the strata of country of origin.

A multilocus genotype test examining whole gene association was performed using step-wise permutation implemented in PLINK.

Microsatellite alleles were tested for association by 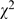 test using the permutation procedure instituted in CLUMP to account for the multiple ways that microsatellite alleles can be combined to form two groups.

## Results

### SNP analyses

SNP rs356220 showed missing genotypes in nearly 18% of controls compared with <1% of cases (*P* = 4.73×10^−15^) suggesting a systematic problem in genotyping of this SNP in control samples, and it was therefore excluded from further analyses. The remaining 31 SNPs were analysed for association with MSA.

Two SNPs showed association with MSA in the NNIPPS samples: rs3822086 (*P* = 0.0044), and rs3775444 (*P* = 0.012) (Table 3). Only rs3822086 survived multiple testing correction for association with MSA (*P* = 0.047). A step-wise whole gene permutation test gave *P* = 0.029 with the same two SNPs retained. We repeated the analysis in the MSA-C subgroup for these two SNPs and found the association strengthened despite more than halving the number of cases: rs3822086 *P* = 0.0024, OR 2.153, (95% CI 1.3–3.6, Breslow-Day test for homogeneity, *P* = 0.81); rs3775444 *P* = 0.0017, OR 4.386 (95% CI 1.6–11.7, Breslow-Day test *P* = 0.36); step-wise whole gene permutation test *P* = 0.0084.

A haplotype of six surrounding SNPs was identified correlating with rs3822086 at r^2^>0.5. Without rs3822086 included, this still showed association at *P* = 0.048, increasing confidence that the association observed was not due to a genotyping artefact. Including rs3822086 strengthened the association with MSA-C. The most strongly associated haplotype, GGATGGT gave *P* = 8.7×10^−4^, omnibus test *P* = 0.027.

As expected, testing of rs3822086 and rs3775444 in PSP showed no association, with unadjusted *P*-values of 0.112 and 0.242 respectively.

### NACP-Rep1 Microsatellite analyses

Permutation-based CLUMP analysis showed no association (*P* = 0.094).

### Replication analyses

The samples from the Institute of Neurology Brain Bank and Paris confirmed association of rs3822086 with MSA (*P* = 0.035) with odds ratio 1.75 (95% C.I. 1.05–2.89).

### Imputation analyses

After imputation of genotypes at ungenotyped SNPs there were a total of 318 SNPs tested, with genotypes predicted at 97.2% accuracy. SNP rs3822086 remained the most strongly associated with MSA. An MSA-associated SNP identified in another study [21], rs11931074, was also associated with MSA in this study (*P* = 0.025). For the MSA-C subgroup, rs3775444 and rs3822086 again remained the most strongly associated SNPs. As for the SNPs in this study, rs11931074 was more strongly associated with MSA-C than with MSA alone (*P* = 0.0125).

## Discussion

We have found two positive associations in the *SNCA* gene with MSA, one with rs3822086 and the other with rs3775444, although only one survives correction for multiple testing. The association is significant because of the role of α-synuclein in the underlying pathology of MSA. Because the phenotypic spectrum of MSA is broad and the relative frequency of cerebellar and parkinsonian presentations appears to be influenced by ethnicity [27] we stratified our cases as MSA-C and MSA-P to explore possible differences in association between these phenotypes in our sample. We observed a stronger association of *SNCA* variants with MSA-C than with MSA as a whole. This is intriguing and requires exploration in other studies, but may indicate that interactions between *SNCA* gene variants and other risk factors influence phenotype in MSA.

Until recently, studies of polymorphisms of *SNCA* had failed to demonstrate any association with MSA either by sequencing the entire coding region in a small number of Japanese cases [11] or by genotyping two microsatellite markers, one in the promoter and the other an intron of *SNCA* in 47 cases from the UK [12], [19]. A study examining SNPs prioritized in a genome-wide association study of Parkinson's disease for follow-up testing in MSA has now identified a strong association of homozygosity for the T allele of SNP rs11931074 and MSA [21]. This SNP has a similar minor allele frequency (0.056) to rs3822086 (0.058) and the two SNPs are in strong linkage disequilibrium (D′ 0.977, r^2^ 0.922). It is therefore not surprising that rs11931074 shows association with both MSA and MSA-C in our study, although the association is not as strong as for rs3822086. It would be of interest to impute rs3822086 in the genome-wide association study samples to see if the stronger association of this SNP persists in that sample set. Nevertheless, this suggests that both rs3822086 and rs11931074 are tagging the same functional genetic variation. Given that both have a relatively low minor allele frequency, this may be a rare haplotype or variant.

The associated variants we have identified are both intronic, and neither is close to a splice site, nor are they predicted to disrupt or alter splicing. They are not in intronic splice enhancer sites, nor alternative cassette exons. They are not especially conserved and are not obviously regulatory. It is therefore likely that a different SNP tagged by these variants is the functional variant, and this is further supported by the haplotypic association we identified for SNPs surrounding rs3822086, which persists even when this SNP is excluded.

There are several factors that could confound this study. For example, population stratification can lead to false positive results. We used three strategies to minimize this. Firstly, the samples were all of European ancestry; secondly we stratified the primary analysis by country of origin; and thirdly we replicated the findings in an independent set of cases. The associated SNP survived multiple testing correction, increasing confidence in the association. Furthermore, the global gene-wise evidence for association was significant and requires no further multiple testing correction making a false positive result less likely. Phenotypic heterogeneity was minimized by using validated clinical criteria with excellent inter-observer agreements, and pathological verification where possible. The possibility of bias inherent in the selection process for enrolment to the NNIPPS study was excluded by testing the MSA-associated SNP for association with the different disease PSP, also using the NNIPPS study samples. PSP is not expected to have an abnormality in the *SNCA* gene since it is not a synucleinopathy: no association was detected. Systematic genotyping errors can lead to false positive results, but we observed strict, comprehensive quality control measures. Furthermore, a haplotype using a set of SNPs surrounding the associated SNP and which correlated with the genotype, also detected the association suggesting that this is not an artefact at the tested SNP.

Our findings show that genetic variation in the α-synuclein gene is associated with sporadic MSA, and suggest that susceptibility to MSA-C may be the greater risk of *SNCA* variation. These results suggest that α-synuclein can play a primary role in the pathogenesis of MSA.

## Supporting Information

Appendix S1 The NNIPPS study Group
(0.04 MB DOC)
