# Supplementary material for: Genetic Variants of the α-Synuclein Gene SNCA Are Associated with Multiple System Atrophy
Source: PLoS One. 2009 Sep 22;4(9):e7114. doi: 10.1371/journal.pone.0007114 (PMC2743996; doi:10.1371/journal.pone.0007114)
Supplement: Appendix S1 — The NNIPPS study Group (0.04 MB DOC) [file pone.0007114.s001.doc]

**Appendix S1: The NNIPPS study Group**

**Co-ordination**: European and UK: P.N. Leigh (London, UK), France: G. Bensimon (Paris, France), Germany : A.C. Ludolph (Ulm, Germany)

**Steering Committee**: Chair : PN. Leigh (London, UK), Members: Y. Agid, G. Bensimon, M. Dib, L. Lacomblez, M. Vidailhet (Paris, France), D. Burn (Newcastle, UK); B. Landwehrmeyer, A.C. Ludolph (Ulm, Germany)

**Independent Data Monitoring and Safety Committee** : Chair : B. Asselain (Paris-France), Members : H. Allain (Rennes, France), D. Chadwick (Liverpool, UK), JE. Perret (Grenoble, France), C. Warlow (Glasgow, UK)

**Technical Committees:**

**Clinical diagnostic criteria:** Chair: D. Burn (Newcastle, UK), Members: Y. Ben-Shlomo (Bristol, UK), AM. Bonnet, J. Fermanian, C. Payan, M. Verny, M. Vidailhet (Paris, France), P. Moore (Liverpool, UK), C. Tranchant (Strasbourg, France)

**Neuro-Pathology:** Chair : J.J. Hauw (Paris, France), Members : C. Duyckaerts, D. Seilhean (Paris, France), S. Al-Sarraj, T. Revesz (London, UK), B. Landwehrmeyer (Ulm, Germany), H. A. Kretzschmar (Munich, Germany)

**Genetics** : Chair: A. Dürr (Paris, France), Members : A. Al-Chalabi (London, UK), N. Wood (London, UK), A. Brice (Paris, France), W. Camu (Montpellier, France), K. Morrison (Birmingham , UK)

**Logistics, Monitoring, Data Management & Statistical analysis:**

Chair : G. Bensimon (Paris, France), **European Project Manager**: M. Graf (Paris, France), **Data Manager**: C. Payan (Paris, France), **Data entry**: P. Paillasseur (Theriamis - St Maur des Fossés, France), **Senior Statistician**: C. Payan (Paris France), Assistant Statistician: H.P. Pham (Paris France), Genetic: A. Al-Chalabi (London, UK), Functional scales development: J. Fermanian (Paris, France), Neuropsychology: R. Brown (London, UK), Health economics: P. Mc Crone (London, UK), **Clinical Research Assistants**: N. Dedise, C. Hermine, S. Sagnes, B. Poître, C. Foucart (Paris, France), A. Dougherty, C. Murphy, H. Mason (London, UK), T. Hermann, K. Klempp, A. Niess, V. Stange (Ulm, Germany), Regulatory affairs France: A. Ouslimani (Paris,

France).

**Principal Investigator France/ Germany/ UK**:

Y. Agid (Paris), A. Ludolph (Ulm), PN Leigh (London).

**Investigators within Countries:**

**France :** F. Viallet, C. Couratier, S. Arguillère  (Aix en Provence); : F. Dubas, C. Fressinaud (Angers) ; L. Rumbach, E. Vidry (Besançon);F. LeDoze, G. Defer, F. Viader, R-M. Marié (Caen); F. Durif, B. Debilly, Ph. Derost, C. Tilignac (Clermont-Ferrand); G. Besson, C. Mallaret (Grenoble); A. Destée, L. Defebvre, (Lille); P. Couratier (Limoges); E. Broussole, H. Mollion (Lyon); JP Azulay, T. Witjas (Marseille); W. Camu, F. Portet, J. Khoris, N. Pageot, G. Garrigues (Montpellier); M. Borg (Nice); M. Vidailhet, S. Sangla (Hôpital St Antoine-Paris), D. Ranoux (Hôpital St Anne-Paris), J.P. Brandel (Hôpital Leopold Belland-Paris), T. De Broucker (Hôpital St Denis-Paris), Y. Agid, B. Dubois, Meininger, Verny, F. Bloch, A..M. Bonnet, L. Lacomblez, D. Maltête, A. Memin, Torni, ML. Welter, J. Worbe (Hopital Pitié-Salpêtrière-Paris), P. Cesaro (Hôpital Henri Mondor-Paris), G. Fenelon (Hôpital Tenon, Paris), (Clinicians); R. Gil, M. Bailbé, S. Venisse, H. Moumy, V. Mesnage, J.L. Houeto, F. Petit (Poitiers); M. Verin (Rennes); C. Tranchant, G. Steinmetz (Strasbourg); C. Prunier, A. Autret, P. Corsia (Tours).

**Germany:** J. Noth, C. Kosinski, C. Geyer, M. Kronenbürger, C. Schlangen (Aachen); K. Einhaeupl, PD G. Arnold, B. Hauptmann, A. Lipp (Berlin); H. Przuntek, T. Müller, G. Gagel-Schweibold, M. Siepmann, S. Benz (Bochum); H. Reichmann, B. Herting (Dresden); C. H. Lücking, I. Bötefür, S. Braune, C. Magerkurth, V. Mylius (Freiburg); S. Zierz, M. Kornhuber, T. Mueller, S. Neudecker, U. Seifert (Halle); R. Dengler, A. Hauswedell, H. Kolbe, T. Peschel, C. Schrader, S. Siggelkow, J. Stewen, H-H. Kapels, C. Winkler (Hannover); C. W. Wallesch, C. Bartels, M. Fork (Magdeburg), T. Brandt, F. Asmus, M. Bauer, T. Gasser, S. Maass, J. Velden, A. Viehöver, D. Wassilowsky, K. Bötzel (München); U. Bogdahn, J. Klucken, Z. Kohl, M. Lange, C. Thun, J. Winkler, B. Winner (Regensburg); R. Benecke, D. Dressler, A. Wolters, G. Zegowitz (Rostock); J. Dichgans, O. Eberhardt, K. Gröschel, T. K. Hauser, J. B. Schulz (Tübingen); A. C. Ludolph, D. Ecker, A. Jung, B. Kramer, G. B. Landwehrmeyer, A. Storch, S.D. Sussmuth (Ulm);

**United Kingdom:** M. Gibson, R. Forbes (Belfast); C.E. Clarke (Birmingham –City Hospital); H. Pall, D Nicholl (Birmingham –Queen Elizabeth Hospital); J. Hodges, T. Bak (Cambridge); P. Moore (Liverpool); PN. Leigh, D. Heaney, C. Blain, S. Azam, V. Williams, J. Isaacs, C. Smallman, B. Stanton (London IoP & GKT); A. Lees, N. Quinn, A. Evans, T. Scaravilli, N. Russo, E. Trikouli, D. Paviour, L. Massey, J Neumann (London NHNN & Queen Square Hospital); P. Newman, Bathgate (Middlesbrough); D. Burn, A. Zermansky, N. Warren (Newcastle upon Tyne); B. Summers (Stafford); C. Counsell (Aberdeen), S. Bhaumick, S. Evans, G. Turner (Guernsey); R. Weiser, C. Lawthom, A. Lowman (Swansea).
